# Imaging dark charge emitters in diamond via carrier-to-photon conversion

**DOI:** 10.1126/sciadv.abl9402

**Published:** 2022-01-07

**Authors:** Artur Lozovoi, Gyorgy Vizkelethy, Edward Bielejec, Carlos A. Meriles

**Affiliations:** 1 Department of Physics, CUNY-City College of New York, New York, NY 10031, USA.; 2 Sandia National Laboratories, Albuquerque, NM 87185, USA.; 3 CUNY-Graduate Center, New York, NY 10016, USA.

## Abstract

The application of color centers in wide-bandgap semiconductors to nanoscale sensing and quantum information processing largely rests on our knowledge of the surrounding crystalline lattice, often obscured by the countless classes of point defects the material can host. Here, we monitor the fluorescence from a negatively charged nitrogen-vacancy (NV^−^) center in diamond as we illuminate its vicinity. Cyclic charge state conversion of neighboring point defects sensitive to the excitation beam leads to a position-dependent stream of photo-generated carriers whose capture by the probe NV^−^ leads to a fluorescence change. This “charge-to-photon” conversion scheme allows us to image other individual point defects surrounding the probe NV, including nonfluorescent “single-charge emitters” that would otherwise remain unnoticed. Given the ubiquity of color center photochromism, this strategy may likely find extensions to material systems other than diamond.

## Teaser

Color centers capturing diffusive photo-induced charge carriers reveal otherwise invisible point defects in a crystal.

## INTRODUCTION

As applications of color centers to precision sensing and quantum information processing continue to grow (*1*), the charge state of a point defect—ultimately defining its spin and optical properties—has emerged as a valuable experimental handle. A nice illustration is the case of the nitrogen-vacancy (NV) center in diamond, where the interplay between spin, photon emission, and charge state has been exploited to demonstrate optical ionization conditional on its starting spin state (*2*). Because the charge state is more resilient to optical illumination than the spin, this “spin-to-charge conversion” (SCC) strategy has, in turn, been used to boost spin detection sensitivity beyond that possible via standard optical readout (*3*–*8*) or to demonstrate long-term spin-state storage robust to laser excitation (*9*, *10*).

Continuous green illumination of an NV leads to cyclic ionization and recharging, thus allowing us to think of the point defect as a local “charge pump” injecting free carriers into the conduction and valence bands as it consecutively changes its charge state from negative to neutral and vice versa (*11*). Because the number of diffusing carriers can be correlated to the defect’s spin state via SCC, this process has been exploited to demonstrate electrical spin readout down to individual NVs (*12*).

Carrier recapture far from the injection site is typically marked by a drastic, long-lived change in the spin and photon emission properties of the trapping defect, meaning that, even in the absence of collecting electrodes, defect-assisted carrier generation and capture can serve as a practical tool, this time to shed light on the microscopic composition of the host crystal. Experiments articulating local laser excitation of NVs followed by nonlocal fluorescence imaging reveal the formation of well-defined charge state patterns, whose response as a function of the excitation laser duration, intensity, and wavelength reveals valuable information on the dynamics of carrier transport and capture (*13*–*15*), including the formation of space charge potentials (*16*). Most recently, these ideas were extended to the limiting case where the free carrier source and/or target trap are formed by individual point defects (*17*, *18*), a geometry that can potentially be exploited to establish a quantum bus between remote qubits (*19*).

Here, we shift the attention back from the carrier capturing defect to the carrier source, which we indirectly image by monitoring the charge state of a probe NV center as we vary the point of laser excitation. Starting with a pair of NVs a few micrometers apart, we first show that single-charge emitters can be revealed individually, a result we attain by working with suitably engineered “islands” formed by few (or single) NVs. We subsequently extend this approach to image more complex color center sets comprising both NV and silicon-vacancy (SiV) centers and show that, unlike the former, the latter do not serve as efficient charge sources under optical excitation in the visible. In the process, we uncover the presence of yet-unseen charge-emitting sites, which we tentatively associate with nonfluorescent vacancy-based complexes.

## RESULTS

### Wavelength-dependent carrier transport between individual color centers

The sample we study in the present experiments has been described in detail before (*15*). Briefly, we use a focused (~1 μm diameter) high-energy accelerator to implant N or Si ions in an electronic-grade diamond crystal (DDK) with a starting nitrogen concentration of ≲5 parts per billion (ppb); the beam energy in either case (20 MeV for N and 45 MeV for Si) corresponds to a depth of ~10 μm, sufficient to rule out potential surface effects in our observations. We use variable ion beam fluences to create islands with different color center content: For nitrogen, the range goes from 1 × 10^8^ ions/cm^2^ (roughly corresponding to ~2 ions per implantation site) to 5× 10^11^ ions/cm^2^, whereas, for silicon, we used greater fluences of up to 5 × 10^13^ ions/cm^2^ so as to compensate for the lower SiV conversion efficiency (*20*–*23*). Upon implantation, we followed a known annealing protocol (*24*) to simultaneously convert nitrogen and silicon atoms into NV and SiV centers. All laser excitation and confocal fluorescence measurements are carried out using a custom-made, multicolor microscope operating under ambient conditions (*8*, *15*).

**Fig. 1.**
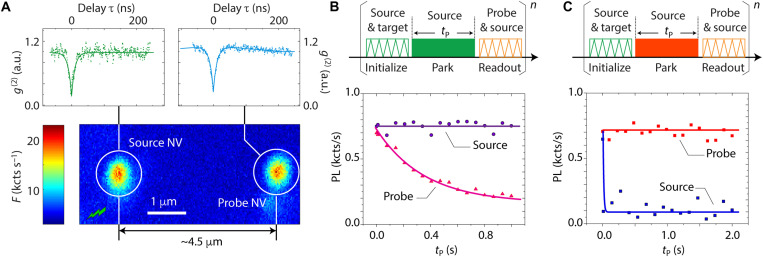
Carrier transport between individual NV centers. (**A**) (Main) Confocal fluorescence image under 520-nm excitation of an NV center pair approximately 4.5 μm apart. (Top insets) Photon autocorrelation curves for each NV; dips below 0.5 confirm the individual nature of each color center. kcts, kilo-counts. (**B**) (Top) Experimental protocol. Zigzags indicate scanning at 520 nm (green) or 594 nm (orange), while the solid rectangle indicates a 520-nm, 1-mW park at the source NV. (Bottom) Observed photoluminescence (PL) at the source and probe NVs as a function of parking time *t*_P_. The probe NV fluorescence gradually decays as the integrated hole capture probability increases with *t*_P_ and conversion from NV^−^ to NV^0^ becomes more likely. (**C**) Same as in (B) except that we park a 632-nm, 1.5-mW laser at the source NV (solid red block). In this case, the source NV quickly ionizes under red illumination, hence preventing additional carrier generation; correspondingly, the charge state of the probe NV remains unperturbed. In (B) and (C), the green (orange) laser power during initialization (readout) is 1 mW (7 μW). a.u., arbitrary units.

The experiments in Fig. 1 lay out our approach: In this first example, we focus on a region of the crystal featuring a grid of single (or near-single) NVs produced by low-fluence N^+^ implantation (1 × 10^8^ ions/cm^2^) and choose two neighboring sites ~4.5 μm apart, each containing individual NVs, which we initialize [with 75% probability (*9*)] in the negatively charged state via green (520 nm) laser excitation. In Fig. 1B, we park the green beam in one of the NVs, the free carrier “source,” for a variable time interval *t*_P_ to induce multiple stochastic cycles of charge state conversion from negative to neutral and back (*9*). Each cycle leads to the injection of a conduction band electron (after NV^−^ ionization) and a valence band hole (upon NV^0^ recombination). Exposed to a stream of freely diffusing carriers, the negatively charged probe NV ultimately converts to neutral as it captures a hole. Note that, despite the coexistence of carriers with opposite signs, Coulombic attraction between the probe NV and the incoming hole makes the NV^−^ to NV^0^ transformation highly one-directional because the converse, though possible, is orders of magnitude less likely (*11*, *15*).

Because red illumination excites NV^−^ but not NV^0^ (whose zero-phonon line amounts to 575 nm), the dynamics at play markedly changes if we use a 632-nm laser during the park (Fig. 1C). Red light quickly ionizes the source NV observed as an immediate fluorescence bleach, with the result that virtually no hole injection takes place; correspondingly, we see no decay in the fluorescence of the probe, whose charge state remains unchanged throughout the laser park duration.

When combined, the experiments in Fig. 1 can be seen as a change in the fluorescence of the probe contingent on the physical properties of the source, emitting carriers or not depending on the excitation wavelength. This correspondence can thus be recast as a “charge-to-photon conversion” (CPC) scheme, which, in principle, can be exploited to expose photoactivated “charge emitters”. To demonstrate the idea, we return to the pair of NVs highlighted in Fig. 1 and generalize the measurement protocol to record the probe NV fluorescence for a variable position of the laser park site, a strategy we hereafter refer to as “CPC imaging.”

**Fig. 2.**
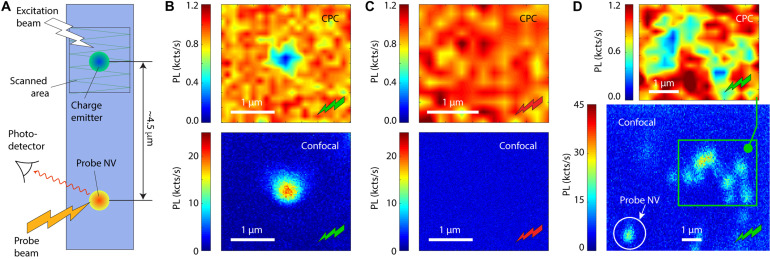
Imaging an individual charge emitter via charge-to-photon conversion. (**A**) Working with the NV pair of Fig. 1A, we generalize the protocols in Fig. 1 (B and C) to monitor the probe NV fluorescence after a laser park at a variable position around the source NV; at each point, the park time is fixed at a value $t_{P}^{\left(0\right)}$ chosen to optimize the image contrast and acquisition time. (**B**) (Top) CPC image of the source NV using the protocol in Fig. 1B for a park duration $t_{P}^{\left(0\right)}=1$ s using 520-nm, 1-mW excitation (green bolt). (Bottom) Direct confocal image of the source NV upon a green scan. (**C**) Same as in (B) but using a 632-nm beam throughout each CPC park and confocal scan (top and bottom images, respectively); the red laser power and park duration during CPC are 1 s and 3.5 mW. (**D**) (Bottom) Confocal image under 520-nm illumination of an NV “island” comprising several NVs; the white circle encloses the probe NV. (Top) CPC image (green laser park; 1 mW, 3 s per point) of the area enclosed in the bottom panel. We observe a good correspondence between the NV and charge emitter positions in the confocal and CPC images. The CPC pixel size is 140 nm in (B) and (D), and 200 nm in (C); CPC images have been smoothed out for clarity.

Figure 2 summarizes the results for a fixed park duration $t_{P}^{\left(0\right)}=1$ s, which we choose so as to optimize the necessary trade-off between the probe fluorescence contrast and the image acquisition time. Because, in this case, the source NV is also a photon emitter, we can use standard confocal microscopy as a reference. Figure 2B compares the results between direct fluorescence readout and CPC imaging of the same single NV using a 520-nm laser throughout the confocal scan or variable position park. We find a one-to-one correspondence, namely, the probe NV fluorescence drops at the location of the source NV but remains unchanged everywhere else. By contrast, we detect no trace of the charge emitter if we move the park laser wavelength from 520 to 632 nm (Fig. 2C); this observation is consistent with the rapid ionization of the source NV under prolonged red excitation, as already seen in Fig. 1.

Given the probabilistic nature of the underlying process and the relatively low hole capture cross sections of neutral or positively charged defects (*15*), CPC imaging can be extended from single charge emitters to groups containing several. This is shown in Fig. 2D, where we implement our strategy in an island with multiple NVs; comparison between the CPC and confocal images—both obtained under 520-nm excitation—shows a nearly perfect correspondence between the NV locations and charge-emitting sites.

### Imaging nonfluorescent charge emitters

**Fig. 3.**
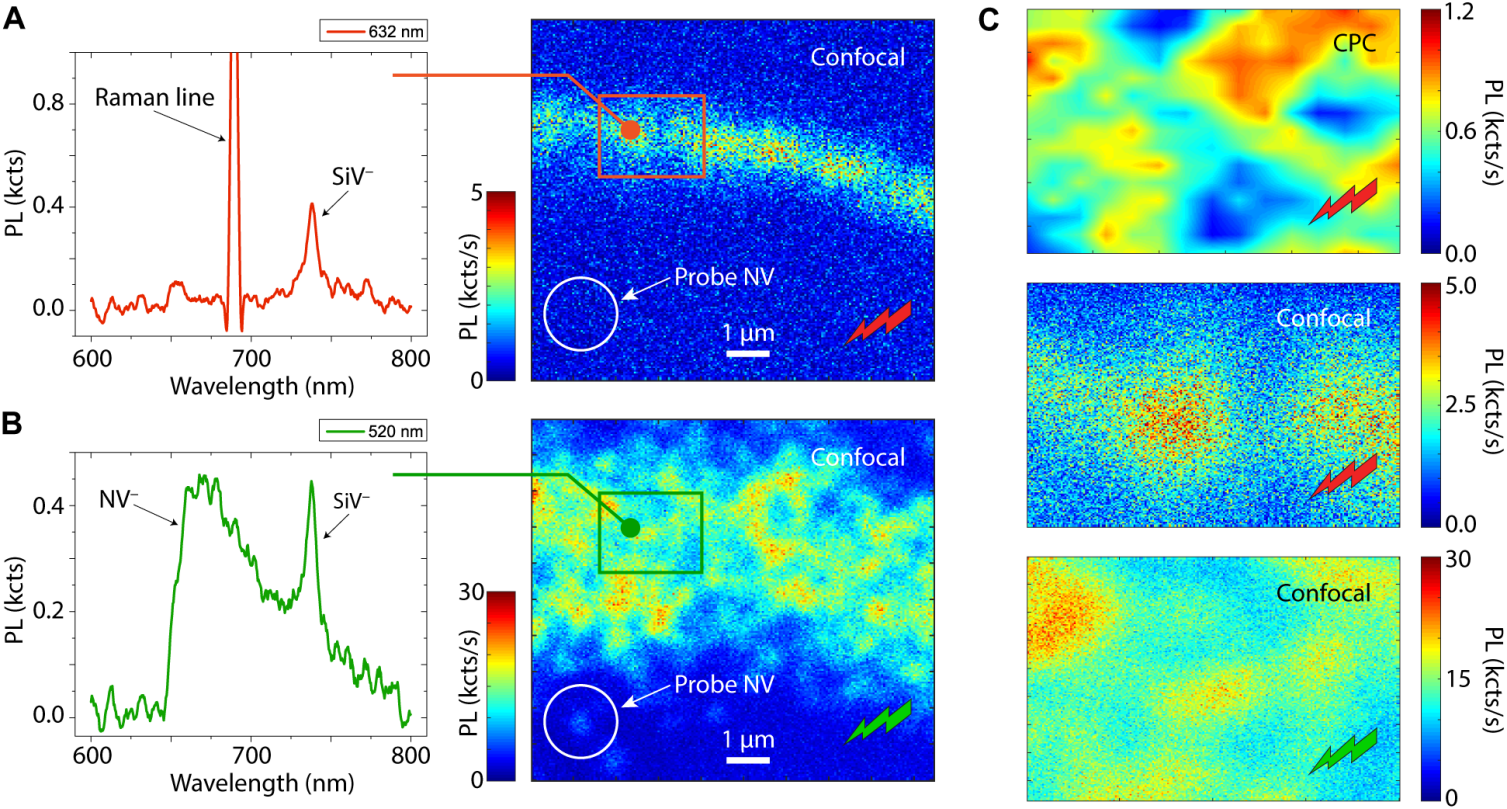
CPC imaging of Si-implanted areas. (**A**) (Main) Confocal image of a Si-implanted region of the diamond crystal under 632-nm excitation. (Left inset) Representative fluorescence spectrum in the bright region of the image; the peak at 737 nm reveals the presence of SiV^−^. (**B**) Same as (A) but for 520-nm excitation. Collateral NV^−^ centers from around the implanted area. The white circle in the lower left corner highlights the probe NV. (**C**) CPC image from a red park (1 s, 4.5 mW per point) and confocal images under red and green excitation of the enclosed area in (A) and (B) (top, middle, and bottom panels, respectively). We find pockets of charge emitters that do not correlate with the positions of SiV^−^ (or NV^−^).

To benchmark our approach in yet more general scenarios, we carried out experiments in a different region of the same diamond crystal, this time in areas implanted with 45 MeV Si ions (also corresponding to a ~10-μm depth). The fluorescence image of Fig. 3A, obtained upon a 632-nm confocal scan, displays the area of interest, namely, a segment within a ~40-μm-diameter circle of implanted Si ions. The presence of negatively charged SiVs is unambiguous, as demonstrated by the 737-nm zero-phonon line in the accompanying fluorescence spectrum (left of Fig. 3A).

Five-hundred-twenty-nanometer confocal microscopy also reveals a broader strip of NV centers spreading to the sides of the Si-implanted area (Fig. 3B). We interpret this observation as a by-product of Si implantation, whose relatively large size leads to multiple vacancies per ion (approximately three to four times more than nitrogen, as calculated by Stopping and Range of Ions in Matter simulations; not shown here for brevity). We surmise that vacancies—whose mobility is relatively high—diffuse during the annealing stage to form NV centers upon association with intrinsic substitutional nitrogen. The concentration of these “collateral” NVs—roughly 1 ppb as deduced from an average over confocal images across the implanted ring—is consistent with the above picture if we take into account the high conversion efficiency of substitutional nitrogen in bulk diamond (of order 50%) (*25*).

Given the large fluence we chose for Si implantation (5 × 10^13^ ions/cm^2^; orders of magnitude greater than for nitrogen, see above), it is hard to imagine that NVs are the only by-product. Figure 3C provides initial evidence in this direction: Here, we rely on a fringe NV [white circle in Fig. 3 (A and B)] to CPC image a segment of the implanted ring containing both SiV and NV centers. We use strong 632-nm illumination during the park, which quickly leads to NV^−^ ionization with (virtually) no hole generation, implying that NV centers in the imaged area remain invisible.

We identify, nonetheless, multiple sites where the probe NV fluorescence bleaches, in the process exposing the presence of red laser–driven pockets of charge emitters. Comparison with the confocal fluorescence image resulting from red excitation—selectively sensitive to SiV^−^ (middle panel in Fig. 3C)—suggests that carriers do not stem from negatively charged SiVs. Photo-induced transformations cycling the charge state between SiV^0^ and SiV^−^—presumably yielding low levels of fluorescence—are still plausible, but because we also identify this class of charge emitters in areas of the crystal presumably devoid of silicon (see below), we deem this possibility unlikely. Furthermore, by examining a 520-nm confocal microscopy image of the same region (bottom panel in Fig. 3C), we find that these charge emitters are mostly located at sites with no NV fluorescence (hence allowing us to rule out unaccounted mechanisms of NV charge emission under red illumination).

These observations are revealing in several ways: SiV centers are known to exist in multiple charge states (*26*–*29*), ranging from SiV^2−^ to SiV^+^, but because their photochromism is poorly understood, it is not a priori clear whether their charge state cycles under red excitation (particularly because red light can excite both SiV^−^ and SiV^0^). On the other hand, the mismatch between the CPC and confocal images—for both SiVs and NVs—hints at a distinct type of point defect, cycling its charge under red excitation without generating fluorescence. We are not aware of prior experiments able to selectively reveal similar fluorescence-free, light-driven charge emitters with diffraction-limited resolution.

**Fig. 4.**
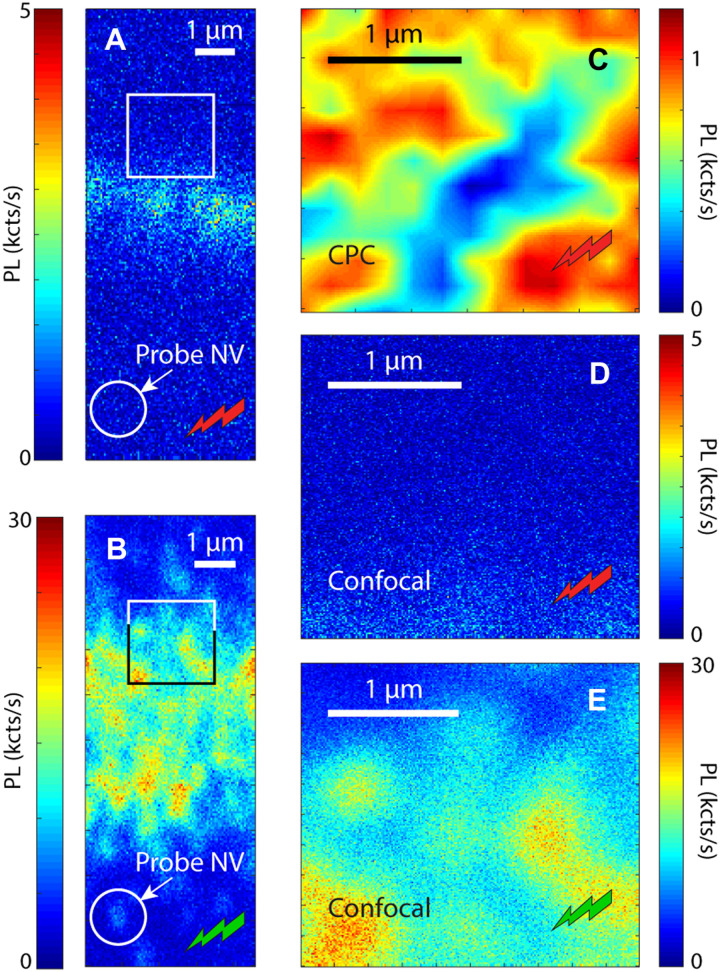
Imaging dark charge emitters. (**A** and **B**) Confocal images under red and green excitation, respectively. The white circle indicates the position of the probe NV. (**C**) CPC image of the enclosed area in (A) under a red laser park. (**D** and **E**) Confocal images of the same area under red and green excitation, respectively.

The results in Fig. 4 confirm and further these ideas: In this case, we move the field of view to an area close to (but away from) the Si implants (Fig. 4, A and B). Confocal microscopy under 632-nm excitation shows that this section of the crystal is virtually devoid of fluorescence (Fig. 4D). CPC imaging, however, shows a completely different landscape, suggesting that dark carrier emitters are widespread; note that, as in Fig. 3C, there is no correlation with the NV sites (see 520-nm confocal microscopy in Fig. 4E), once again ruling out this color center as the carrier source under red light.

**Fig. 5.**
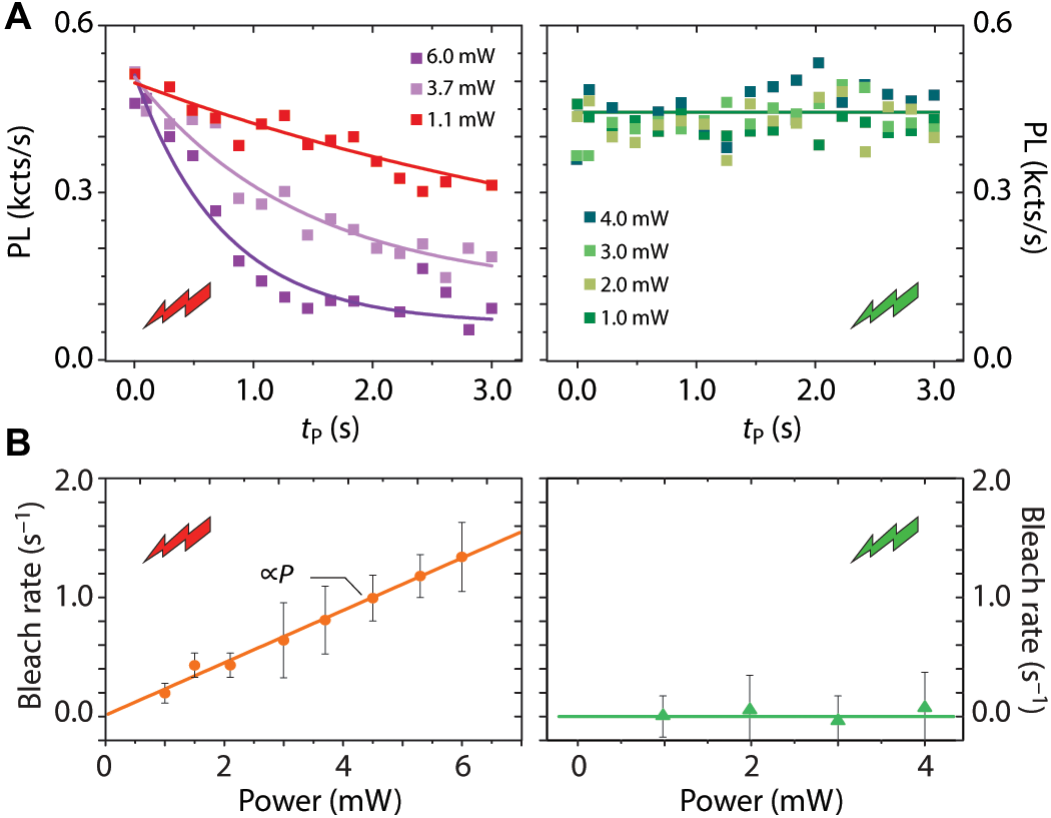
Nonfluorescent charge emitter response at different wavelengths. (**A**) Probe NV photoluminescence as a function of the park power on a dark charge emitter 7 μm away. In these experiments, we apply the protocol in Fig. 1C (red park; left) or Fig. 1B (green park; right) for various laser powers. Solid lines are exponential fits. (**B**) Probe NV photoionization rate as a function of the applied park laser power using red or green excitation (left and right panels, respectively). Solid lines are linear fits.

Without attempting an in-depth examination of the type of emitter at play, beyond the scope of this article, CPC detection can provide some initial clues: This is shown in Fig. 5, where we park the laser beam at a site known to host a charge emitter but with no SiV or NV fluorescence and monitor a probe NV 7 μm away as a function of time for different red laser intensities. From the linear relation between the NV fluorescence bleach rate and the applied laser power [see left panels in Fig. 5 (A and B)], we infer that ionization and recharging of the source charge emitter is a one-photon process, somewhat reminiscent of that inferred from observations in other chemical vapor deposition diamonds (*30*). We find almost no change in the probe NV fluorescence upon a green laser park at the same site [right panels in Fig. 5 (A and B)], indicating that the charge state cycling stops at shorter wavelengths. Note that, while less intuitive, this behavior is also seen in other emitters such as the NV [whose charge state interconversion becomes inefficient when illuminated with blue light (*9*)].

Although the information presently at hand is insufficient to ascertain the microscopic nature of these dark emitters, the observation of collateral, Si implantation–induced NVs suggests the concomitant formation of vacancy-related complexes, some of which we can already examine in light of the above constraints. Concrete illustrations are the V (*31*) and N_2_V (*32*–*34*) centers, the latter arguably stemming from the binding of a vacancy with a pair of adjacent substitutional nitrogens (the symbol V denotes diamond vacancy). Both defect types are known to exhibit photochromism (*35*) between the negative and neutral charge states, but interconversion under red illumination seems unlikely owing to the unfavorable energy scales at play (V^−^ and N_2_V^−^ show zero-phonon lines at 394 and 503 nm, respectively). Given the many alternative possibilities, however, additional work will be needed to more closely examine other candidate photochromic defects, such as complexes integrating nitrogens and protons (*36*–*40*), which cannot be ruled out yet.

## DISCUSSION

In summary, using an NV center as a charge probe, we demonstrated the ability to image individual or small groups of NVs micrometers away via changes in the probe fluorescence stemming from the capture of photo-generated holes. Applying this approach to areas of the diamond crystal previously implanted with Si ions, we found that SiV centers do not undergo photochromism under red excitation. These same observations, however, revealed the presence of previously unseen pockets of nonfluorescent point defects presumably undergoing charge state cycles of ionization and recombination under red illumination (but not green). The physical nature of these charge emitters is presently unclear, although the use of CPC detection already imposes some constraints on future models. In this vein, we see CPC as a technique akin to photoluminescence excitation spectroscopy (PLE), both reporting on the integrated number of quantum particles—charge carriers or photons—produced by a source under illumination of a given color. Correspondingly, extending the above results to systematically monitor the system response as a function of the excitation wavelength would amount to spectrally fingerprinting the carrier emitter. The latter, in turn, could be exploited to separate different classes of point defects very much as in PLE.

Given the ubiquity of photochromism and carrier capture, it is reasonable to assume that the present approach will also find use in the investigation of other semiconductor platforms of interest. An immediate example is the case of silicon carbide, known to host color centers whose charge properties are similar to those found in diamond (*41*–*43*). Another complementary direction is the combined application of subdiffraction techniques such as Stimulated emission depletion microscopy (STED) (*44*, *45*) so as to ionize and image carrier capture with higher spatial resolution down to ~10 nm. Whether or not this enhanced form of CPC can be applied to monitor photoactivated charge transport in macromolecules is an intriguing question worth investigating in further detail. Last, while short-range carrier capture is likely to render photocurrent detection considerably less efficient (particularly if the collecting electrodes are ~10 μm apart or more), the use of external fields might prove, nonetheless, rewarding as a strategy to shed light on carrier propagation and/or alter its capture probability.

## MATERIALS AND METHODS

We carry out all experiments using a multicolor confocal microscope with an oil immersion objective (numerical aperture = 1.3) adapted to focus all incoming beams and collect the sample photoluminescence. Green (520 nm), red (632 nm), and orange (594 nm) lasers are coupled into a single-mode fiber to ensure a similar beam intensity profile and best possible overlap of the excitation volumes. A combination of long-pass, short-pass, and band-pass optical filters is used when separating the photoluminescence from SiV^−^ and NV^−^. We read out the NV charge state by scanning an area around the probe NV with a low-power (7 μW) 594-nm orange excitation beam and then integrate the photoluminescence from the pixels corresponding to the position of the probe NV. By slightly increasing the integrated area of the scan, we can account for potential instabilities in the sample position, which otherwise would have compromised the contrast in the single-point-illumination readout.

At high red laser power and/or short distances between the target area and the probe NV, it is possible to bleach the probe NV^−^ photoluminescence due to ionization caused by the red beam tail and by scattered red light. This hinders the bleaching caused by hole capture and is therefore unwanted in the CPC protocol. We circumvent this effect by recording reference bleaching curves where the target is at the same distance from the probe NV but in a section of the crystal with no charge emitters. CPC-compatible conditions are those where no bleaching is observed (the case in all our experiments).
